# Transmission dynamics study of tuberculosis isolates with whole genome sequencing in southern Sweden

**DOI:** 10.1038/s41598-019-39971-z

**Published:** 2019-03-20

**Authors:** Nader Alaridah, Erika Tång Hallbäck, Jeanette Tångrot, Niclas Winqvist, Erik Sturegård, Kerstin Florén-Johansson, Bodil Jönsson, Erik Tenland, Christina Welinder-Olsson, Patrik Medstrand, Bertil Kaijser, Gabriela Godaly

**Affiliations:** 1 Laboratory medicine, Department of Microbiology, Immunology and Glycobiology, Lund University, Lund, Sweden; 2 Regional Office for Infectious Disease Control and Prevention, Malmö, Sweden; 3 Translational Medicine, Clinical Infection medicine, Lund University, Lund, Sweden; 4 National Bioinformatics Infrastructure Sweden (NBIS), SciLifeLab, Department of Molecular Biology, Computational Life Science Cluster, Umeå University, Umeå, Sweden; 5 Department of Infectious Diseases, Sahlgrenska Academy, University of Gothenburg, Gothenburg, Sweden; 6 Translational medicine, Department of Clinical Virology, Lund University, Malmö, Sweden

## Abstract

Epidemiological contact tracing complemented with genotyping of clinical *Mycobacterium tuberculosis* isolates is important for understanding disease transmission. In Sweden, tuberculosis (TB) is mostly reported in migrant and homeless where epidemiologic contact tracing could pose a problem. This study compared epidemiologic linking with genotyping in a low burden country. *Mycobacterium tuberculosis* isolates (n = 93) collected at Scania University Hospital in Southern Sweden were analysed with the standard genotyping method mycobacterial interspersed repetitive units-variable number tandem repeats (MIRU-VNTR) and the results were compared with whole genome sequencing (WGS). Using a maximum of twelve single nucleotide polymorphisms (SNPs) as the upper threshold of genomic relatedness noted among hosts, we identified 18 clusters with WGS comprising 52 patients with overall pairwise genetic maximum distances ranging from zero to nine SNPs. MIRU-VNTR and WGS clustered the same isolates, although the distribution differed depending on MIRU-VNTR limitations. Both genotyping techniques identified clusters where epidemiologic linking was insufficient, although WGS had higher correlation with epidemiologic data. To summarize, WGS provided better resolution of transmission than MIRU-VNTR in a setting with low TB incidence. WGS predicted epidemiologic links better which could consolidate and correct the epidemiologically linked cases, avoiding thus false clustering.

## Introduction

Tuberculosis (TB) remains a major public health issue in European countries with 323,000 new cases per year^1^. In 2017, 533 new tuberculosis patients were reported in Sweden, which was a decrease by 6,4% compared to the top year 2015 with influx of asylum seekers from high tuberculosis incidence countries, i.e. Somalia, Eritrea, Afghanistan and Ethiopia^2^. Sweden is by all means a low incidence country, as the rest of the Nordic countries, with 5.3 tuberculosis cases per 100,000 inhabitants. Most of the infected patients are between 10 to 29 years of age and foreign born, of which this group constituted approximately 86% of the new cases the past years^2^. To control and prevent disease spreading, epidemiologic tracing with personal communication is the basis, followed by genotyping of the isolated strains. Three genotyping methods were traditionally used before 2016, i.e. restriction fragment length polymorphism (RFLP) and a *Mycobacterium tuberculosis*-specific multiple locus variable number of tandem repeat (MIRU-VNTR), combined with spoligotyping^3–5^. In Sweden, MIRU-VNTR was set as the new standard since 2012 by the Public Health Agency of Sweden, to which *M*. *tuberculosis*-complex isolates were sent for genotyping. There are some limitations to MIRU-VNTR, as this technique may not distinguish between closely related genotypes and may be suboptimal among immigrants from countries with high incidence of TB, where genetically closely related strains circulate over extended periods of time^6,7^. Genetic mutations in these strains accumulate, resulting thus often in pairwise SNP distances of $>$12, but the MIRU-VNTR typing pattern may not change and could wrongly be interpreted as recent transmission in the country of immigration^8^.

Whole genome sequencing (WGS) is now rapidly becoming the standard for typing *M*. *tuberculosis* isolates as this technique provides increased resolution over MIRU-VNTR-based clustering and is considered to be superior in defining the extent and direction of tuberculosis transmission^9–14^. However, most of the initial studies were from high TB incidence settings, while less is known about this technique in low TB incidence countries. Recently, WGS analysis on outbreak of isoniazid-resistant TB in London showed that this technique may not be useful in identifying the direction of transmission as previously reported^15^. Other studies from low TB incidence countries showed that WGS is comparable to MIRU-VNTR in native patients, as opposed to foreign-born patients where standard genotyping overestimates recent TB transmission^13^. In this study, we reanalysed *M*. *tuberculosis* strains isolated during the years 2004–2014 with WGS in order to compare the different genotype techniques in a low TB incidence country.

## Material and Methods

### Study design and population

We sequenced 100 isolates of *M*. *tuberculosis* from an archive of 801 frozen cultures obtained between 2004 and 2014 that is held at the Regional Mycobacterial Reference Laboratory for Scania County, Sweden. We selected isolates to estimate genomic diversity within and between hosts. TB is a mandatory notifiable infectious disease to the public health agency of Sweden^2^ and relevant diagnostic codes, microbiology results and contact tracing were obtained from the Scania county office for infectious disease prevention and control.

### Epidemiologic investigation and Traditional genotyping of bacterial strains

*M*. *tuberculosis* isolates were cultured at SUS Malmö. 24-loci MIRU-VNTR was performed on 93 laboratory-confirmed, culture-positive isolates at the clinical microbiology laboratory at Sahlgrenska university hospital, Gothenburg, Sweden. Clinical, demographic, and microbiological data were available for each isolate. We obtained epidemiological data from the Scania county office for infectious disease prevention and control. Sweden guidelines for contact tracing recommend screening of household contacts for every new index case, together with at-risk individuals as well as any other pointed contacts in the community if the index case is thought to be infectious.

### Bacterial isolates - selection and DNA extraction

Retrospectively, all notified TB cases with *M*. *tuberculosis* culture positive samples were identified and stored at −80 C. A total of 100 strains were grown on Lowenstein-Jensen medium for up to 4 weeks in a Biosafety level 3 laboratory. Then approximately one single colony was suspended in 0.5x TE buffer and heat inactivated at 80 C for 20 min before we could sonicate the samples at 35 KHz for 10 min and genomic DNA was extracted using QIAamp DNA Mini Kit (Qiagen**)** in a Biosafety level 2 laboratory.

### Genomic DNA sequencing and library preparation

Sequencing libraries were prepared from 100 ng DNA using the TruSeq Nano DNA sample preparation kit (cat#FC-121,4001/4002 Illumina inc.) according to the manufacturer’s instructions guide (#15041110, HiSeq 2500 system) by SNP&SEQ Technology Platform at Uppsala University. Seven isolates were excluded due to low genomic DNA quantity and quality and the remaining 93 samples were queued for sequencing on 1 lane PE125 and generated 236 M read pair. Raw reads were archived under accession number (to be determined).

### Identification, Annotation, and Confirmation of SNPs

Raw reads were trimmed with Trimmomatic [Bolger, A.M., Lohse, M. and Usadel, B (2014) Trimmomatic: A flexible trimmer for Illumina sequence data. Bioinformatics 30:15, 2114–2120] to remove adapter sequence and low-quality bases. Only reads at least 36 bases long after trimming were kept. Trimmed reads were mapped to the reference genome CDC1551 (NCBI accession GCA_000008585.1_ASM858v1) using the program BWA mem (http://bio-bwa.sourceforge.net/)^16,17^, version 0.7.13. The median depth of sequencing was (124.7x), with an average of 99.55% of the reference genome being covered by at least one read after quality control and trimming of reads. The program BWA mem (http://bio-bwa.sourceforge.net/)^16,17^, version 0.7.13, was used to map the trimmed reads to the reference genome followed by variant calling. Variants were called using freebayes (https://github.com/ekg/freebayes), version 1.0.2. A coverage of at least 8, an alternate allele count of at least two, and an alternate fraction of at least 20% was required to evaluate a position. We only retained positions with phred-scaled quality score of ≥Q20 using vcftools (https://vcftools.github.io/index.html)^18^. The codon sequences for all single nucleotide polymorphisms (SNPs) positions are extracted using information from annotation with SnpEff^19^.

The bioinformatics software CLC Genomics Workbench 11.0, Qiagen, was used to study the five largest clusters which comprise more than three patient-isolates. After trimming, the sequences were mapped to the reference genome CDC 1551 (NCBI Reference Sequence: NC_002755.2, assembly accession GCA_000008585.1), followed by a restrict calling to target regions of InDels and structural variants, as well as a Local Realignment. Single nucleotide variants (SNP) were analysed and called for by a Fixed Ploidy Variant Detection step. Fixed ploidy variant parameters- Ploidy: 2, Required variant probability 90%. Variant detection and general filters- Minimum coverage: 30, Minimum count: 10 and Minimum frequency: 20%.

### Phylogenetic analyses and strain diversity

Phylogenetic analysis of the single nucleotide polymorphisms (SNPs) was conducted for both the entire set of 93 isolates as well as for four additional genomes/assemblies (CCDC5180 (East Asian lineage 2), EAI/OSDD271 (East African-Indian lineage 3), Haarlem and CTRI-2 (Euro-American lineage 4) that represent the modern *M*. *tuberculosis* lineage. These four genomes were aligned to the reference genome CDC1551 using the nucmer program in the MUMmer package, version 3.23^20^ and SNPs were extracted using the show-snps program in the same package. The resulting lists of SNPs were merged into a vcf-like file and then combined with the variants from the 93 samples using GATK CombineVariants. SNPs located within 12 bp of each other, SNPs in PE/PPE/PGRS genes phage, repeat and transposons were excluded to avoid any concern about errors in the read alignment in those repetitive regions of the genome^8^. Furthermore, SNPs in additional drug-resistance associated genes^21^ were also removed to exclude the possibility that homoplasy of drug resistance mutations would significantly decrease the reliability of phylogeny^22,23^. Ambiguous positions were also excluded and then concatenated alignment was used to generate a midpoint rooted phylogenetic tree in RAxML (version 7.3.4) under GTRCAT substitution model with 100 bootstrap replicates.

The Microbial Genomics Module, CLC, was used to create a Maximum Likelihood (ML) SNP-tree for the five largest clusters, comprising 26 patient isolates. In this analysis the minimum coverage in each sample = 10, minimum coverage percentage of average required = 10, prune distance = 10, minimum z-score = 1,96 and a bootstrap analysis performed with 600 replicates. A MIRU-VNTR phylogenetic tree was performed using BioNumerics v.7.6/Applied Maths (a BioMerieux Company).

### Genomic cluster analysis

Genomic clusters were determined independently of the epidemiological data and a cluster was defined if no more than 12 SNPs separated a patient isolate from that of at least one isolate from another patient in the cluster. Twelve SNPs was previously defined as the upper threshold of genomic relatedness noted within hosts and between related hosts^10^.

### Statistical analysis

Prism 6 f for Mac OS X was used for statistical analysis. The Chi-square test was used to analyse differences between patients clustered by WGS and patients that were not; Fisher’s exact test was used for cell counts below five. Significance was accepted at *p* < 0.05, *p* < 0.01, or *p* < 0.001.

### Ethical approval

The research was approved by the Medical Ethics Committee at the Lund University, Lund, Sweden (Dnr. 2014/2). The study was approved by the Regional Ethical Review Board (Dnr 2014/2), Lund University, Sweden. All experiments were performed in accordance with relevant guidelines and regulations.

## Results

### Study selection

**Figure 1:**
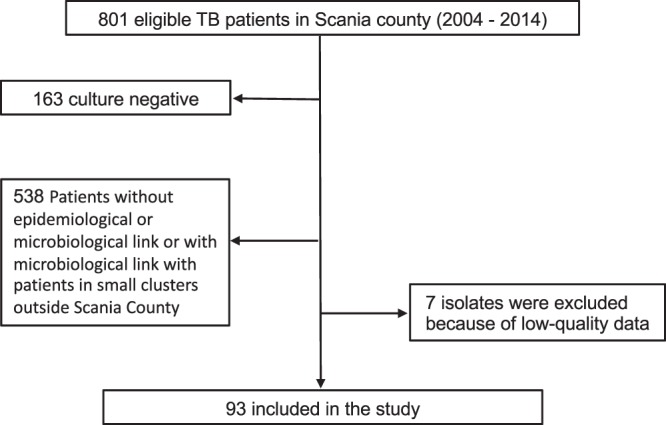
Flow chart of sample collection. Graph show number of isolates/sequences included in the study.

The study population consisted of isolates from 801 eligible TB patients treated between 2004 and 2014 at four infectious disease clinics and one paediatric clinic in Scania County, Sweden. In total, 100 isolates were selected for WGS. The remaining 701 isolates were excluded since they were either culture negative, had no epidemiological or microbiological connection at all, or with an epidemiological connection in small clusters outside Scania County. Of the remaining 100 patient *M*. *tuberculosis* isolates, we excluded seven isolates due to insufficient DNA quantity and quality (Fig. 1). The final 93 isolates were selected for WGS due to epidemiological connections in pairs or groups. The selected sample thus contained patient isolates from potential clusters with clinical relevance for contact tracing. The phylogenetic tree revealed that three of the seven main global lineages of *M*. *tuberculosis* circulated in Scania county during the time of the sampling (Supplemented Fig. 1) [36,49–51]. The vast majority of isolates belonged to lineage 4 (62%) (Europe, America and Africa) while 30% belonged to lineage 2 (East Asia), with lesser representation from lineage 3 (8%, India and East Africa).

### Clinical characteristics

The majority of the patients were adults at the time of the diagnosis (median age 39 years), presenting the disease as pulmonary tuberculosis (76%), extra pulmonary TB (20%) or both simultaneously TB (4%) (Table 1). The age range was 1 to 91 years, of which two patients were infants. None of the patients were positive for the human immunodeficiency virus (HIV). Clinical outcomes were recorded through December 2016, with a minimum of 12 months of follow-up for all patients. The majority of patients had favourable outcomes; 84 patients (91%) were cured or completed the treatment. Five patients (5%) did not complete the treatment, and one patient (1%) had a relapse of pulmonary TB. Three TB related deaths (3% of patients) were recorded during the follow-up period. The mean age of the clustered cases was 40 years (range 1–91) compared to 41 years (range 1–87) of the WGS un-clustered cases. There was no statistical difference between the clustered cases and the un-clustered cases regarding the parameters in Table 1, except in two cases. All homeless patients were found in the clustered group (p = 0.0021), while most newly arrived immigrants were found in the un-clustered group (p = 0.0200). There was no statistical difference in lineage association between clustered and un-clustered patients (lineage 4: 54% vs 69% (p < 0.1373), lineage 2: 34% vs 27% (p = 0.4997) and lineage 3: 12% vs 4% (p = 0.2342)).

**Table 1:** Characteristics of 93 Patients with Laboratory-Confirmed Tuberculosis and epidemiologic link in Scania region-Sweden between 2003 and 2014 overall and comparing clustered vs un-clustered.

Characteristics	All patients (n = 93)	Clustered (n = 52)	Un-clustered (n = 41)
Age–(years)			
Mean (±Standard deviation)	40.6 (±19.4)	39.3 (±18.2)	42.4 (±21.7)
Range	1–91	1–91	1–87
Gender			
Male	57 (61)	32 (62)	25 (61)
Female	36 (39)	20 (38)	16 (39)
Place of birth			
Swedish born	28 (30)	15 (29)	13 (32)
Foreign born			
Somalia	17 (18)	7 (13)	10 (24)
Vietnam	8 (9)	4 (8)	4 (10)
Others	40 (43)	26 (50)	14 (34)
Tuberculosis Type			
Pulmonary	71 (76)	44 (85)	30 (73)
Extra pulmonary	19 (20)	9 (17)	10 (24)
Both	3 (4)	1 (2)	2 (5)
Smear positive			
Yes	59 (60)	36 (69)	23 (56)
No	34 (37)	16 (31)	18 (44)
Treatment Outcome			
Completed treatment	84 (90)	47 (90)	37 (90)
Incomplete treatment	5 (5)	2 (4)	3 (7)
TB-related Death	3 (3)	2 (4)	1 (2)
Relapse	1 (1)	1 (2)	0 (0)
Risk Factors			
History of TB exposure	4 (4)	2 (4)	2 (5)
Immunosuppressive Rx	5 (5)	2 (4)	3 (7)
Homelessness	10 (11)	10 (19)	0 (0)
Newly arrived Immigrants	10 (11)	2 (4)	8 (20)

^a^Data are n (%) or mean ± Standard Deviation on WGS analysed patients.

### Transmission clusters

**Figure 2:**
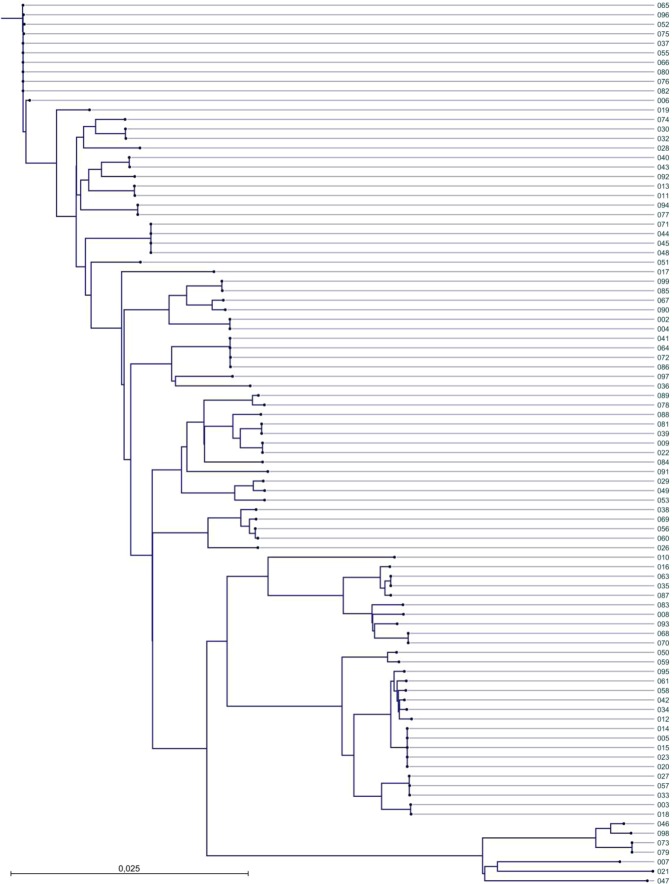
Phylogenetic tree constructed by pairwise distance (maximum likelihood) of 93 *Mycobacterium tuberculosis* isolates including CDC1551 as reference genome.

WGS and MIRU-VNTR was performed on the 93 *M*. *tuberculosis* isolates. MIRU-VNTR typing and pattern analysis resulted in 19 clusters comprising 59 patients. Five clusters comprised more than three patients, while the remaining 14 clusters comprised of less than three patients (Table 2, Fig. 2). These 19 MIRU-VNTR determined clusters comprised of 59 of the 93 patients and corresponded to a clustering proportion of 63**%**.

**Table 2:** Transmission clustering.

Cluster	Epidemiological link	VNTR	WGS
1	037, 052, 055, 065, 076, 080, 082, 096 and uncertain	**006**, 037, 052, 055, 065, **066**, **075**, 076, 080, 082, 096	037, 052, 055, 065, **006**, **075**, 076, 080, 082, 096
2	044, 045, 048, 071	044, 045, 048, 071	044, 045, 048, 071
3	041, 064, 072, 086	041, 064, 072, 086	041, 064, 072, 086
4	**027**, 033, 057	033, 057	**027**, 033, 057
5	Uncertain	005, **012**, 014, 015, 020, **034**, **049**, **061**, **095**	005, 014, 015, 020, **023**
6	040, 043	040, 043	040, 043
7	**067**, **090**	067, 090	No connection
8	2,004	2,004	2,004
9	039, 081	039, 081	039, 081
10	056, 060	056, 060, **069**	056, 060
11	003, 018	003, 018	003, 018
12	068, 070	068, 070	068, 070
13	073, 079	073, 079	073, 079
14	011, 013	011, 013	011, 013
15	030, 032	030, 032	030, 032
16	009, 022	009, 022	009, 022
17	Uncertain	077, 094	077, 094
18	Uncertain	085, 099	085, 099
19	035, 063	035, **016**	035, 063
20	**007**, **010**	No connection	No connection
21	**036**, **046**	No connection	No connection

^*^Bold = technique that made the original clustering.

Using a maximum of twelve SNPs as the upper threshold of genomic relatedness noted among hosts^10^, we identified 18 clusters with WGS comprising 52 patients with an overall pairwise genetic maximum distances ranging from zero to nine SNPs (median 2.5) (Table 2, Fig. 2). Five of the clusters comprised of three patients or more, while the remaining 14 clusters comprised of less than three patients. These 18 WGS transmission clusters corresponded to a clustering proportion of 56**%**.

Eleven of the clusters involved only foreign-born patients, but none of the clusters involved only Swedish-born patients (Table 1). However, in the largest cluster, which comprised 10 patients, 9 were Swedish-born and one was born in Somalia. The Swedish-born patients belonged to a community of homeless people with alcohol abuse at the time of diagnosis. Interestingly, the Somalian patient did not belong to the homeless community, and in contrast with the native patients, had TB in a lymph node. The time range between the first and last isolate in this cluster was 7 years (Table 1).

### Cluster similarities and differences between MIRU-VNTR and WGS

WGS clustered the same isolates as MIRU-VNTR (Table 2). However, there was a major difference in cluster 5 and interesting diversities in clusters 1, 4, **7**, 10 and 19. In cluster 5, MIRU-VNTR grouped additional five strains compared to WGS, while WGS included one new strain compared to MIRU-VNTR. Analysis of the phylogenetic tree revealed that the additional strains in cluster 5 added by MIRU-VNTR were 88 SNPs apart for isolate 012, 77 SNPs apart for isolate 034, 76 SNPs for isolate 061 and 72 SNPs for isolate 095. Isolate 049 was calculated to differ 826 SNPs from the rest of WGS cluster 5, hence this strain was probably given the wrong MIRU-VNTR type from the beginning. Similar results were found for the differences in clusters 1, **7**, 10 and 19. Interestingly, WGS identified additional strains in clusters 4, 5 and 19 compared with MIRU-VNTR, and one additional strain was identified with MIRU-VNTR compared with WGS analysis in cluster 1. This difference can be explained by the fact that MIRU-VNTR is a 24-locus based molecular typing method, while WGS covers single nucleotide polymorphisms across the entire *M*. *tuberculosis*-genome (approximately 4,4 M bases). The SNP-analysis method is therefore much more discriminative and covers regions that might otherwise be missed, hence providing a more accurate picture of real closeness of strains.

**Figure 3:**
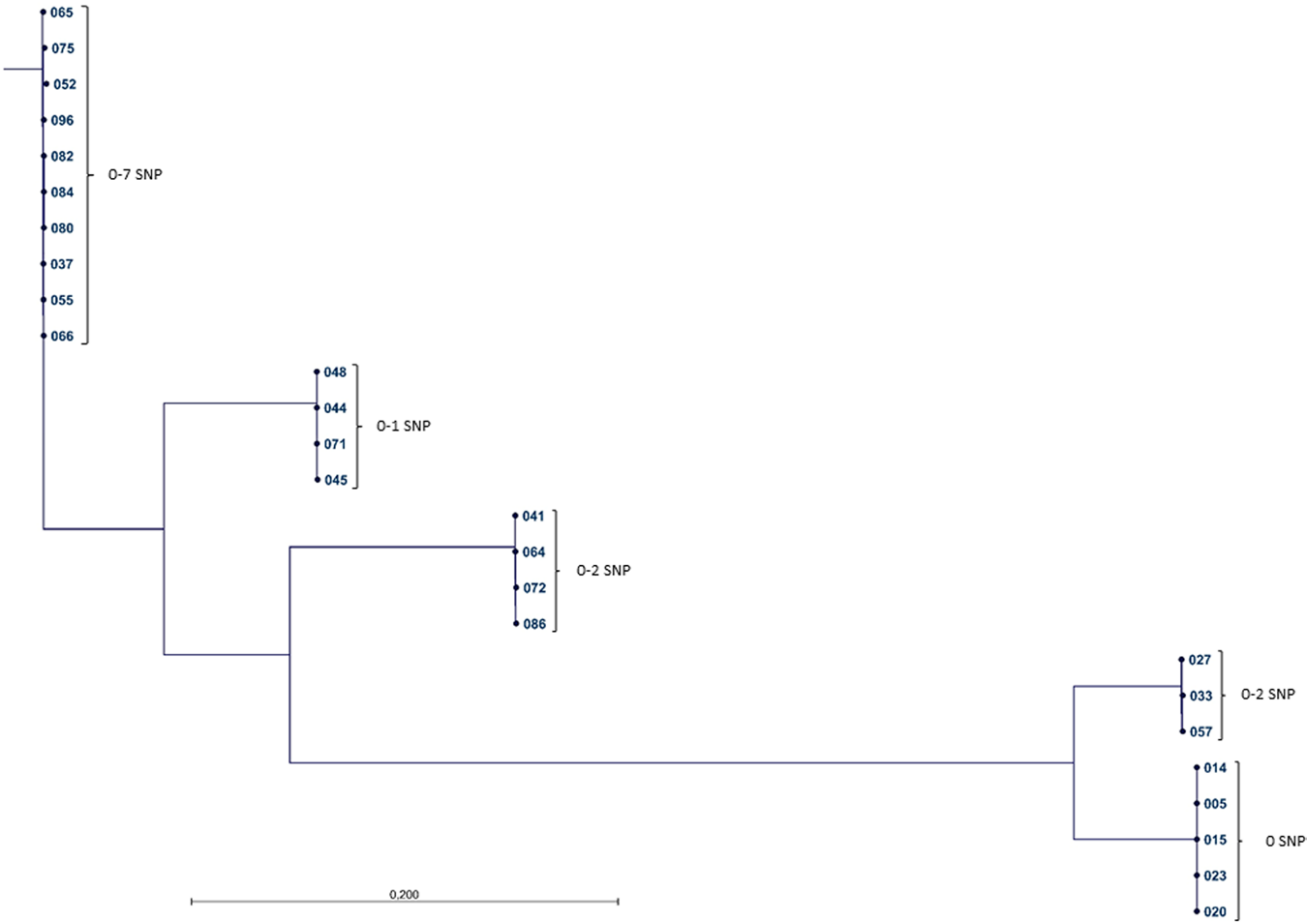
ML SNP tree with CLC. A ML SNP-tree of the five largest clusters in the study, comprising of 26 patient isolates was created using the reference genome CDC1551. Within the four smaller clusters the strains differ by 0–2 SNP in a genetic pairwise comparison. In the largest cluster the patient isolates are separated by 0–7 SNP.

Further analysis of the five largest clusters revealed that all of the *M*. *tuberculosis*-isolates were separated by 0–7 SNP, except for patient isolate no. 06, which differed 16–18 SNP in a pairwise comparison. This strain had the same VNTR pattern as the other isolates in the cluster, but no epidemiological contact tracing data were found to support transmission, why this isolate was removed. The isolates of the remaining four clusters differed by only 0–2 SNP in a pairwise comparison in their corresponding groups (Fig. 3).

### Correlation with epidemiologic investigation

Epidemiological contact tracing complemented with *M*. *tuberculosis* genotyping is considered to be important for the understanding of transmissions^15^. Interviews of patients identified 18 possible clusters of which 3 clusters comprised more than three patients (Fig. 2, Table 2). Contact tracing identified 18 clusters comprising 47 patients. Of these, two clusters with two patients in each were removed as they were not supported by MIRU-VNTR and WGS. Interestingly, WGS supported the epidemiological data more accurately and had a higher correlation with epidemiological data than the MIRU-VNTR. Our study revealed that epidemiologic tracing had an 86% sensitivity and 92% specificity using WGS. The sensitivity and specificity of MIRU-VNTR versus WGS were 96% and 80% respectively. The MIRU-VNTR method thus showed more false positive connections while epidemiologic tracing showed more false negative connections.

### Geographic and genetic distances within molecular clusters

The 43 patients found in contact tracing represented 16 transmission events that all had ≤12 SNPs genetic distance (range 1–11 SNPs). Of the two epidemiologically linked clusters (010 and 007, 036 and 046), patient strains were separated by more than 12 SNPs and were excluded (Figs 2 and 3). Eight of 59 (13.5%) of the MIRU-VNTR linked patients (006, 012, 034, 049, 061, 095, 069, 016) were separated by more than 12 SNPs and were thus excluded by WGS. The genetic distance for events between patients that could not be epidemiologically linked was up to more than 1300 SNPs; 0–2 SNPs for the seven patients that lacked confirming epidemiological data but that were clustered by WGS (i.e. 066, 075, 005, 014, 015, 020, 023), and approximately 700–1300 SNPs for 36 of the 93 non-epidemiologically linked patients that were not clustered by WGS (007, 008, 010, 012, 016, 017, 019, 021, 026, 027, 028, 029, 033, 036, 038, 042, 046, 047, 050, 051, 057, 058, 059, 069, 074, 078, 083, 084, 087, 088, 089, 091,0 92, 093, 097, 098).

The ability of genomic clustering to identify unclear outbreaks was most evident in the large clusters. In the largest cluster, seven SNPs or fewer separated the 11 patients with a background of homelessness for whom contact tracing had been difficult (Fig. 3). The ability to rule out transmission was particularly evident in the second largest MIRU-VNTR cluster, in which isolates (012, 034, 049, 061 and 095) from recent immigrants from Somalia, shared same pattern with the other patients but were separated with more than 12 SNPs. In this cluster, isolates from five smear negative patients, with no known epidemiological link, could not be genetically linked by ≤12 SNPs, either (Fig. 2).

## Discussion

Four hospitals in Scania has Sweden’s third largest uptake area of new TB cases. 93 TB patients whom were previously analysed by traditional genotyping techniques were included in this study. Compared to the standard genotyping and epidemiologic tracing, WGS had an overall high match in identifying cluster transmissions in this patient population. Analysing the two genotyping techniques MIRU-VNTR and WGS, we observed that MIRU-VNTR clustered eight additional patients compared to WGS. This is in good agreement with other studies who state that MIRU-VNTR generally overestimates transmission of *M*. *tuberculosis* in countries with low incidence of TB^24,25^. Our results are also in good agreement with the study of Wampade *et al*. stating that MIRU-VNTR typing may be suboptimal among immigrants from countries with high incidence of TB, where genetically closely related strains circulate over extended periods of time^7^. As an example, cluster 5 in our study comprises an immigrant group from Somalia, where both MIRU-VNTR and WGS clustered these patients. However, while WGS clustered five patients, MIRU-VNTR included additional five patients and missed one. The same is true for clusters 10 and 19, with patients from Ethiopia and Vietnam. The diversity in results between the two methods could be explained by the different base of genetical analysis. WGS provides a more accurate picture of the real closeness between strains.

Native cases of TB in Sweden occur especially in subpopulations with alcohol and/or drug abuse, homelessness and poor living conditions as well as individuals of old age. In these populations, endemic strains, responsible for the TB outbreaks, might be frequent, but epidemiologic contact tracing could pose a problem. In contrast, the foreign born are usually identified through health checkups and through contact tracing, but the country of infection could be hard to identify as well as to measure the effect of TB control in Sweden^26^. In our study, cluster 1 consisted of native homeless patients sampled 2007–2013, where most of the patients had pulmonary TB, compared to an immigrant group from Somalia sampled 2004–2005 comprising cluster 5, with diverse disease locations from joints, pulmonary and pleura. Interestingly, epidemiologic tracing identified the domestic patients, but missed the Somalian patient in cluster 1 and all Somalian patients in cluster 5. This could be due to the diversity in infection location in the Somalian patients making the epidemiologic linking difficult.

Advances in next generation sequencing technology have provided a whole new chapter in informative epidemiology and WGS is now a verified technique for investigation of various aspects of TB^22^. Currently there is no international standard for the SNP distance cut-off to rule in a possible transmission, and various cut-offs have been applied in studies in different countries, which limits the ability to compare data^11,27–30^. The upper threshold of 12-SNP distance to identify transmission was proposed by Walker *et al*. based on studies in two low incidence TB settings, but this limit has been used in other studies as well^10,31–33^. TB-isolates with a common strain identity should be alike and differ by a few number of SNPs in a pair-wise comparison. A strength in our study is the high coverage between epidemiologic links and WGS. A cut-off of 12 SNPs was also valid in our study in Sweden, a low incidence country. The limit of 12 SNPs is the upper limit and valid only if combined with confirming clinical epidemiological data. Walkers studies furthermore state that isolates separated by five or fewer SNPs are likely a result of recent transmission^31^. WGS was initially shown to provide increased resolution over MIRU-VNTR-based clustering^31^, but this technique was recently proven to be insufficient in fully resolving the chains of transmission^15^. An explanation could be that multiple transmissions can occur with no detectable SNP acquisition, and identical isolate pairs cannot be proven to have resulted from a recent transmission even without supporting epidemiological evidence. International guidelines for clustering cut-offs are however still needed to ensure comparison between different studies.

A limitation of our study is that we determined the sequence of the patient’s dominant genotype, but this approach is recommended as isolating single colonies prior to sequencing is likely lead to overestimation of the SNPs between cases resulting from direct transmission^15^. Compared to other studies, we analysed four of the modern *M*. *tuberculosis* strains in order to reveal geographic distribution^13^. Different *M*. *tuberculosis* strain lineages have also been associated with variable virulence, transmissibility, disease phenotypes and drug resistance profiles^34^. Interestingly, we found that the vast majority of isolates belonged to lineage 4, suggesting American/European/Middle Eastern origin^35,36^. Another difference to other studies is that we have used *M*. *tuberculosis* CDC1551 as a reference strain, due to its continued passage in humans, in comparison to *M*. *tuberculosis* H37Rv that has been passaged *in vitro* for many years^37,38^. Also, there is a benefit of using CDC1551 as a reference in WGS for mapping of resistance genes and variant calling, as H37Rv is known for some deletions in its genome^39^.

To summarize, we conclude that WGS is well suited for identifying transmission clusters in settings with low TB incidence. However, as a future goal an international standardization is needed on all factors to ensure that WGS data are comparable between different studies.

## Supplementary information

Supplementary Fig 1
