# Supplementary material for: Transmission dynamics study of tuberculosis isolates with whole genome sequencing in southern Sweden
Source: Sci Rep. 2019 Mar 20;9:4931. doi: 10.1038/s41598-019-39971-z (PMC6426893; doi:10.1038/s41598-019-39971-z)
Supplement: Supplementary file 1 — Supplementary Fig 1 [file 41598_2019_39971_MOESM1_ESM.pdf]

Transmission dynamics study of tuberculosis isolates with whole genome sequencing in southern Sweden

Nader Alaridah[1], Erika Tång Hallbäck[5], Jeanette Tångrot[4], Niclas Winqvist[2,3], Erik Sturegård[3], Kerstin Florén-Johansson[5], Bodil Jönsson[5], Erik Tenland[1], Christina Welinder-Olsson[5], Patrik Medstrand[6], Bertil Kaijser[5], Gabriela Godaly[1]

[1]Laboratory medicine, Department of Microbiology, Immunology and Glycobiology, Lund University, Lund, Sweden. [2]Regional Office for Infectious Disease Control and Prevention, Malmö, Sweden. [3]Translational Medicine, Clinical Infection medicine, Lund University, Sweden, [4]National Bioinformatics Infrastructure Sweden (NBIS), SciLifeLab, Department of Molecular Biology, Computational Life Science Cluster, Umeå University, Sweden, [5]Department of Infectious Diseases, Sahlgrenska Academy, University of Gothenburg, Gothenburg, Sweden. [6]Translational medicine, Department of Clinical Virology, Lund University, Malmö, Sweden.

Supplemented Figure 1. Phylogenetic tree showing that four of the seven main global lineages of *M. tuberculosis* circulated in Scania county during the time of the sampling. 62 % of the isolates belonged to lineage 4 (Europe, America and Africa) and 30 % belonged to lineage 2 (East Asia), with lesser representation from lineage 3 (8 %, India and East Africa).

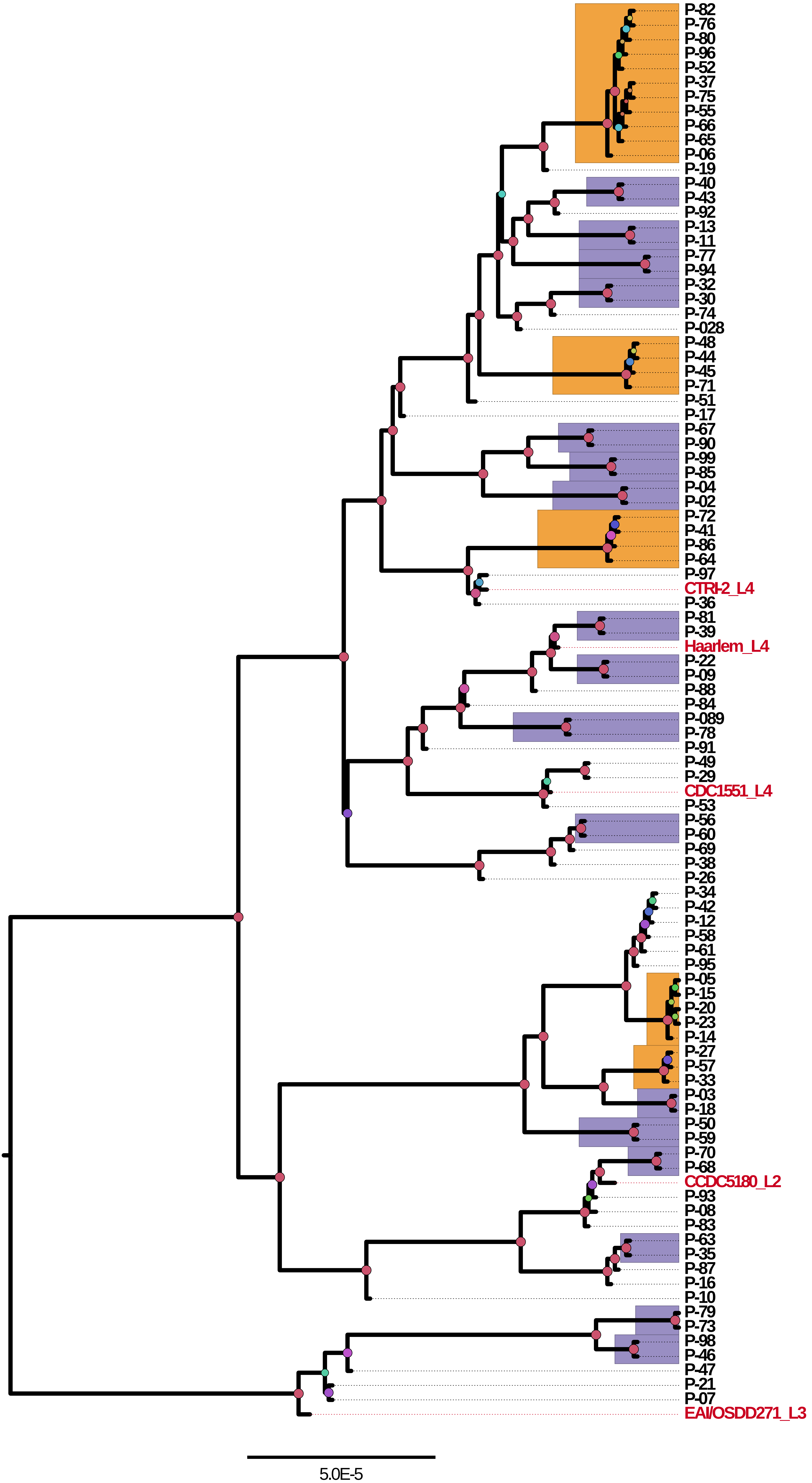
P-82
P-76
P-80
P-96
P-52
P-37
P-75
P-55
P-66
P-65
P-06
P-19
P-40
P-43
P-92
P-13
P-11
P-77
P-94
P-32
P-30
P-74
P-028
P-48
P-44
P-45
P-71
P-51
P-17
P-67
P-90
P-99
P-85
P-04
P-02
P-72
P-41
P-86
P-64
P-97
CTRI-2_L4
P-36
P-81
P-39
Haarlem_L4
P-22
P-09
P-88
P-84
P-089
P-78
P-91
P-49
P-29
CDC1551_L4
P-53
P-56
P-60
P-69
P-38
P-26
P-34
P-42
P-12
P-58
P-61
P-95
P-05
P-15
P-20
P-23
P-14
P-27
P-57
P-33
P-03
P-18
P-50
P-59
P-70
P-68
CCDC5180_L2
P-93
P-08
P-83
P-63
P-35
P-87
P-16
P-10
P-79
P-73
P-98
P-46
P-47
P-21
P-07
EAI/OSDD271_L3
5.0E-5
